# Supplementary material for: Pancancer Analysis of the Oncogenic and Prognostic Role of NOL7: A Potential Target for Carcinogenesis and Survival
Source: Int J Mol Sci. 2022 Aug 25;23(17):9611. doi: 10.3390/ijms23179611 (PMC9455868; doi:10.3390/ijms23179611)
Supplement: Supplementary file 1 [file ijms-23-09611-s001.zip › ijms-1855853-supplementary.pdf]

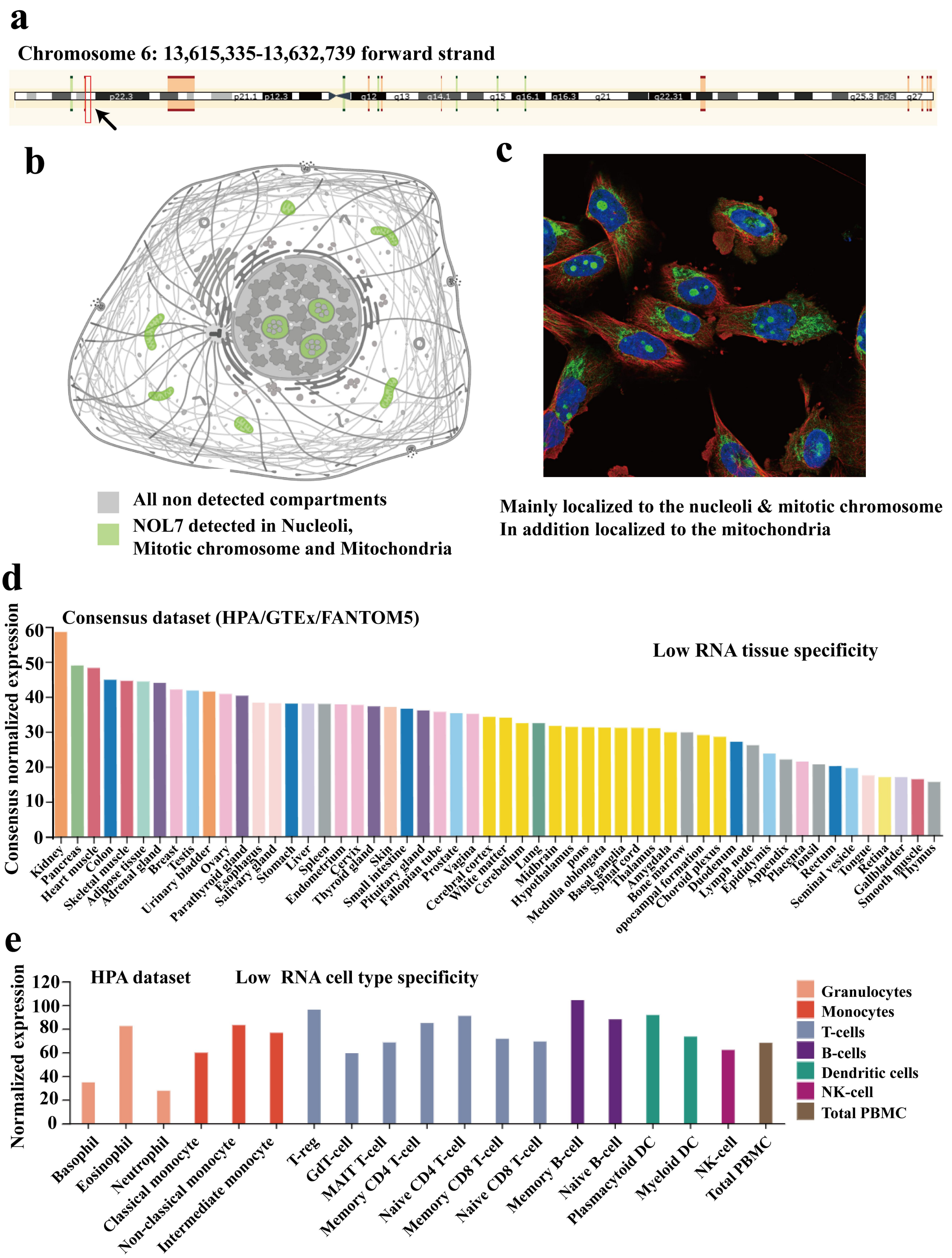

Supplementary Figure S1. Expression level of *NOL7* in different normal tissues and blood cells. **(a)** Genomic location of human *NOL7*. **(b-c)** Location of *NOL7* protein in eukaryocyte. **(d)** *NOL7* gene expression in different normal tissues using the consensus datasets of HPA, GTEx and FANTOM5. **(e)** *NOL7* gene expression in different normal blood cells using the consensus datasets of HPA, Monaco and Schmiedel.

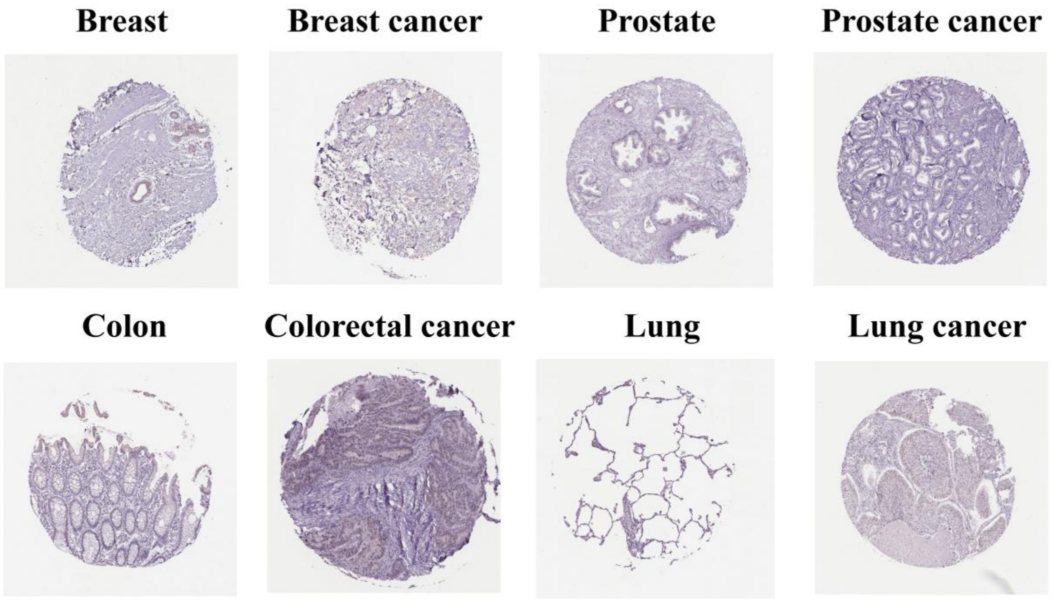

Supplementary Figure S2. Immunohistochemistry images of *NOL7* protein in tumor tissues including breast cancer, prostate cancer, colon cancer, and lung cancer, and corresponding normal tissues, obtained from HAP dataset.

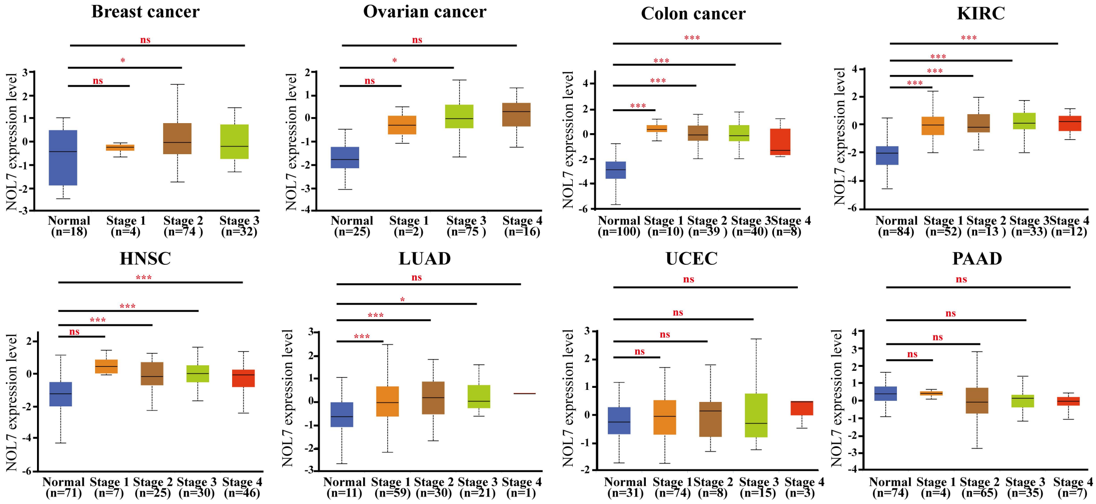

Supplementary Figure S3. Correlation between *NOL7* protein expression and clinicopathology of various cancer types including breast cancer, ovarian cancer, colon cancer, KIRC, HNSC, LUAD, UCEC, and PAAD. * $p < 0.05$; ** $p < 0.01$; *** $p < 0.001$.

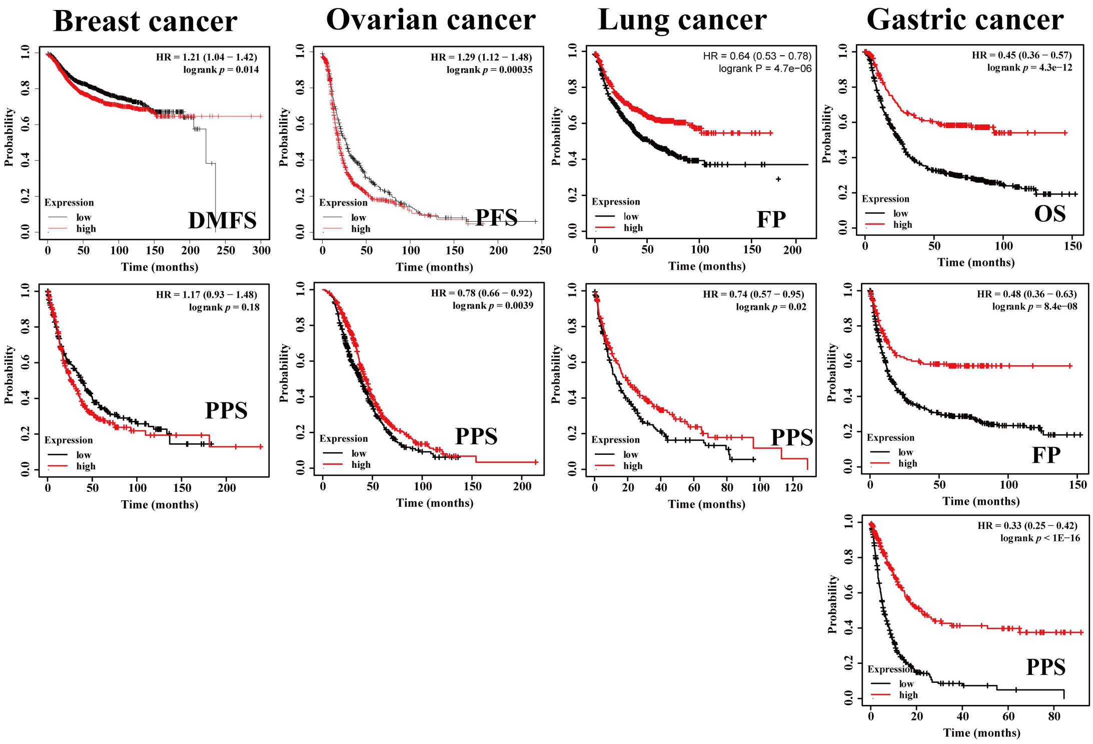

Supplementary Figure S4. Correlation between *NOL7* gene expression and survival prognosis of tumors in TCGA dataset, analyzed in Kaplan-Meier plotter. DMFS: distant metastasis free survival, PFS: progression free survival, FP: first progression, PPS: post progression survival post progression survival.

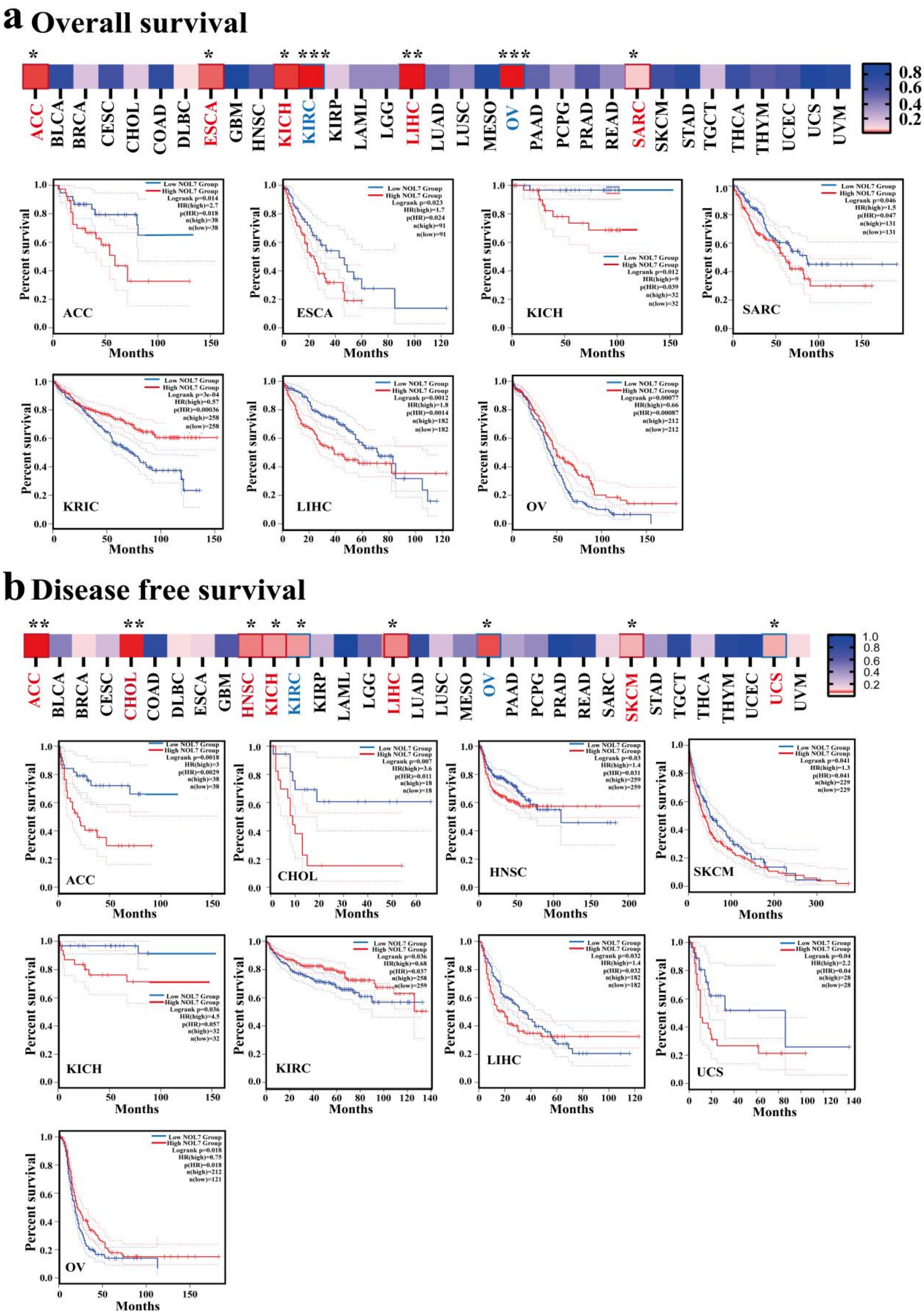

Supplementary Figure S5. Association between the *NOL7* expression and the OS **(a)** and DFS **(b)** of patients in different cancer types, as examined using GEPIA2. * $p < 0.05$; ** $p < 0.01$; *** $p < 0.001$.

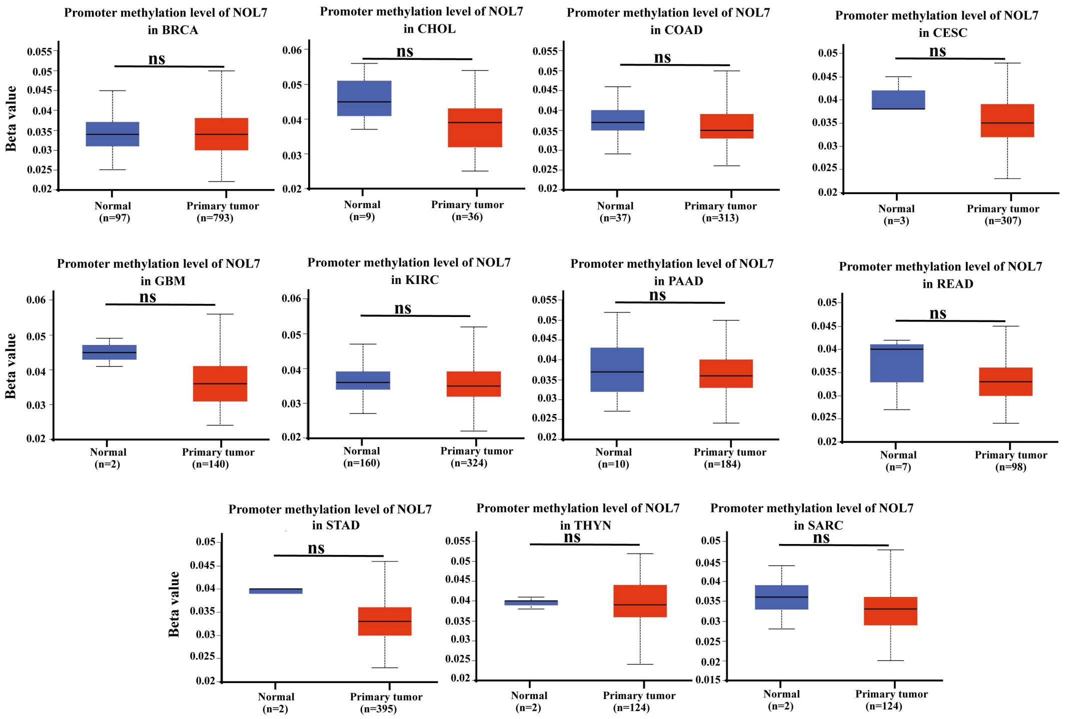

Supplementary Figure S6. No significant difference of promoter methylation level of *NOL7* between primary tumors and normarl tissues was observed in BRCA, CHOL, COAD, CESC, GBM, KIRC, PAAD, READ, STAD, and THYN. ns, No significance.

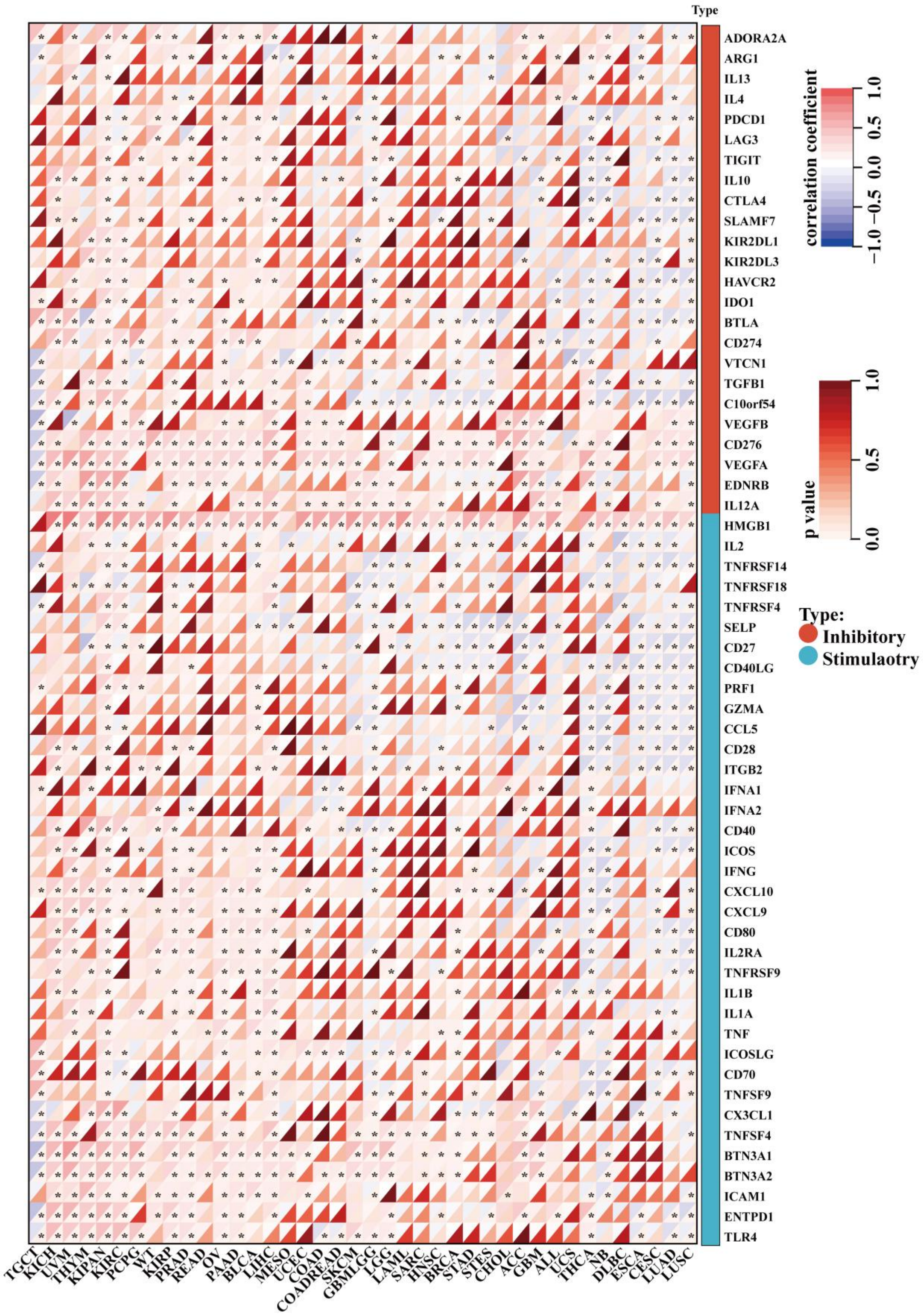

Supplementary Figure S7. Association between *NOL7* expression and immune-related cytokines and immune checkpoint gene expression across 40 TCGA tumors. * $p < 0.05$; ** $p < 0.01$; *** $p < 0.001$.

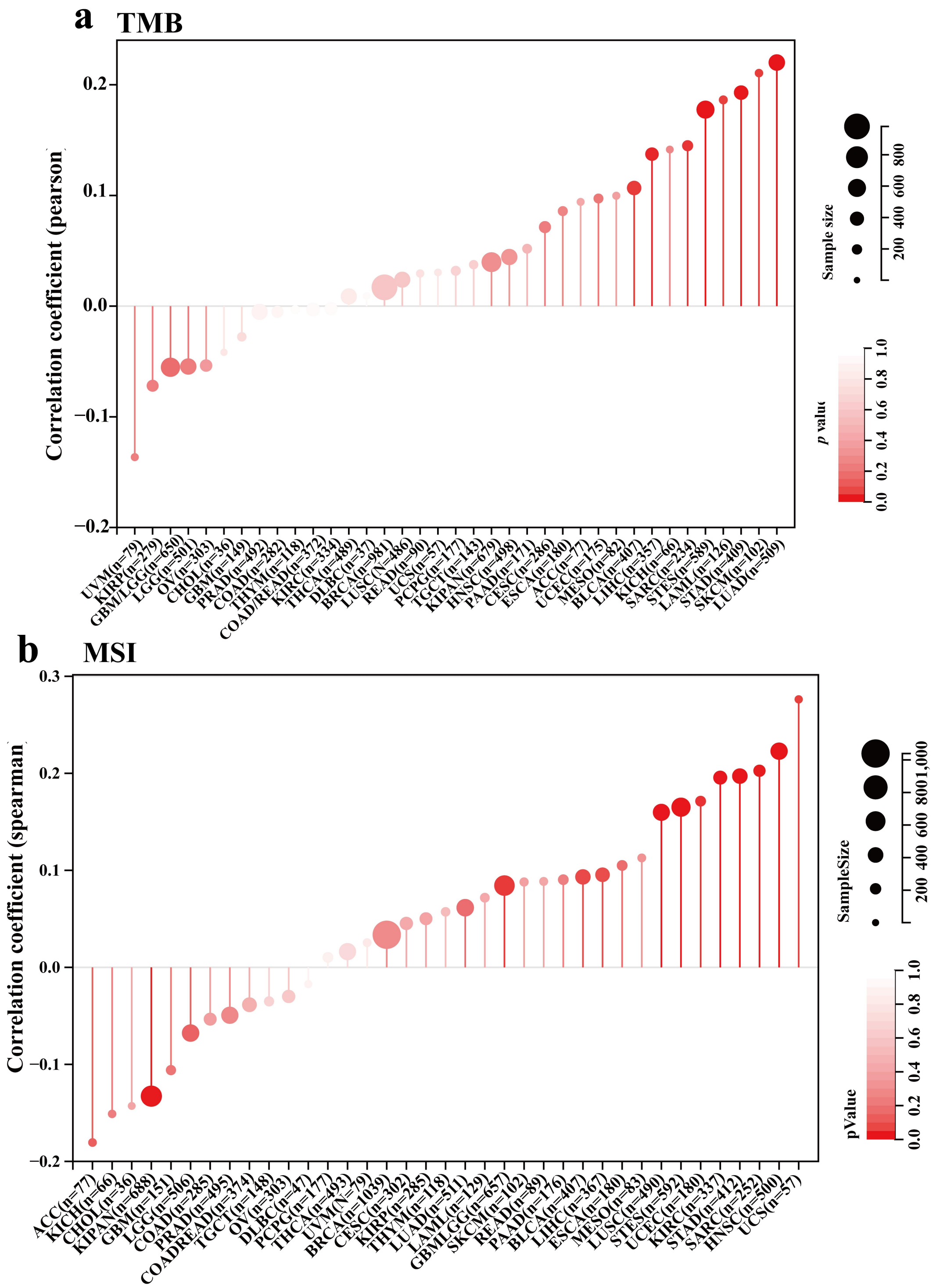

Supplementary Figure S8. Correlation between *NOL7* expression and **(a)** tumor mutational burden (TMB) and **(b)** microsatellite instability (MSI) in different tumors of TCGA dataset.

Factor	Subgroup	Sample size	OS		RFS		DMFS		PSS	
			HR	*p*	HR	*p*	HR	*p*	HR	*p*
**ER status-IHC**	ER positive	3499	1.52	0.0082	1.35	0.00011	1.43	0.0094	1.51	0.028
	ER negative	2168	0.71	0.05	0.97	0.8	0.86	0.27	1.11	0.68
**ER status-array**	ER positive	5526	1.2	0.12	1.46	9.1e-10	1.21	0.046	1.12	0.42
	ER negative	2009	0.7	0.031	0.96	0.71	0.84	0.2	0.93	0.74
**TP53 status**	mutated	272	0.7	0.32	0.68	0.12	0.51	0.083	0.99	0.98
	Wild type	388	0.84	0.59	1.07	0.76	1.44	0.32	0.83	0.59
**PR status**	PR positive	1559	2.53	0.022	1.2	0.006	1.31	0.24	1.85	0.25
	PR negative	1989	0.81	0.39	1.13	0.29	0.87	0.36	0.55	0.25
**HER2 status**	HER2 positive	1273	1.27	0.19	1.26	0.037	1.1	0.59	1.16	0.51
	HER2 negative	6262	1.19	0.12	1.49	6.1e-12	1.24	0.016	1.13	0.38
**Subtype-StGallen**	basal	1494	0.57	0.0044	0.97	0.81	0.86	0.36	0.66	0.15
	Luminal A	3511	1.28	0.12	1.61	1.3e-08	1.22	0.13	1.17	0.38
	Luminal B	2015	1.23	0.25	1.42	8.9e-05	1.25	0.12	0.93	0.76
	HER2+	515	1.06	0.84	1.03	0.86	0.83	0.47	1.25	0.56
**Subtype - PAM50**	basal	1671	0.79	0.23	1.19	0.11	0.96	0.77	0.6	0.072
	Luminal A	2470	1.38	0.13	1.43	0.00078	1.23	0.21	1.13	0.61
	Luminal B	2005	0.88	0.46	1.19	0.049	0.97	0.86	1.04	0.87
	HER2+	1080	0.93	0.73	1.1	0.45	1	1	1.16	0.55
	normal	309	0.83	0.71	1.29	0.46	0.8	0.64	NA	NA
**Lymph node status**	Lymph node positive	2153	1.02	0.92	1.22	0.023	0.99	0.92	1.06	0.78
	Lymph node negative	2829	1.33	0.096	1.32	0.00069	1.44	0.0033	1.43	0.083
**Grade**	Grade 1	576	1.48	0.37	1.52	0.1	2.49	0.035	1.35	0.55
	Grade 2	1795	1.2	0.37	1.38	0.004	1.41	0.02	1.12	0.64
	Grade 3	2058	0.86	0.32	1.02	0.81	0.81	0.12	1.04	0.85
**Pletenpol subtype**	Basal-like 1	418	0.44	0.045	1.18	0.45	0.88	0.65	0.4	0.17
	Basal-like 2	165	0.42	0.1	0.93	0.82	0.92	0.82	NA	NA
	immunomodulatory	462	0.58	0.21	0.93	0.74	0.92	0.76	0.98	0.98
	mesenchymal	382	0.94	0.86	1.03	0.9	0.75	0.35	0.75	0.52
	Mesenchymal stem-like	201	0.82	0.71	0.57	0.12	0.47	0.16	NA	NA
	Luminal androgen receptor	413	0.95	0.87	0.98	0.91	0.82	0.51	0.75	1.15

Table S1. Subgroup analysis on the correlation of NOL7 expression and prognosis of breast cancer cases.
HR, hazard ratio; OS, overall survival; RFS,relapse free survival; DMFS, distant metastasis free survival;
ER, Estrogen receptor; PR, Progresterone receptor; HER2, human epidermal growth factor receptor-2
TP53,Tumor Protein P53; NA, not available data; *p* value less than 0.05 is shown in red.

Factor	Subgroup	Sample size	OS		FP		PPS	
			HR	*p*	HR	*p*	HR	*p*
**Histology**	adenocarcinoma	865	0.64	0.00015	0.78	0.11	0.64	0.058
	Squamous cell carcinoma	675	1.13	0.33	0.88	0.64	0.66	0.43
**gender**	female	817	0.82	0.1	0.64	0.0022	0.76	0.15
	male	1387	0.9	0.21	0.64	7e-04	0.7	0.0412
**Smoking history**	Exclude those never smoked	970	0.81	0.045	0.81	0.093	0.75	0.054
	Only those never smoked	247	0.33	0.00029	0.52	0.008	0.51	0.035
**Stage**	Stage I	652	0.84	0.22	0.86	0.49	0.69	0.22
	Stage II	320	0.69	0.044	0.98	0.94	0.46	0.019
	Stage III	70	1.49	0.15	NA	NA	NA	NA
**grade**	Grade I	202	0.81	0.27	0.88	0.55	0.98	0.93
	Grade II	310	0.9	0.5	1.34	0.17	1.16	0.54
	Grade III	77	1.15	0.68	1.42	0.39	0.8	0.68
**AJCC stage t**	T1	475	1.1	0.5	1.57	0.085	1.01	0.97
	T2	686	0.98	0.89	0.8	0.15	0.96	0.8
	T3	99	0.96	0.88	1.13	0.8	NA	NA
	T4	48	1.29	0.44	NA	NA	NA	NA
**AJCC stage n**	N0	863	1.1	0.39	1.15	0.39	0.99	0.95
	N1	296	1.05	0.75	0.86	0.53	0.98	0.93
	N2	113	0.97	0.89	1.88	0.065	1.13	0.74
**AJCC stage m**	N0	818	1.17	0.13	0.8	0.39	0.78	0.43
**surgery**	Only surgical margins negative	730	0.59	1e-05	0.46	1.8e-09	0.83	0.22
**radiotherapy**	NO	276	0.98	0.92	1.35	0.12	1.11	0.65
	YES	73	0.83	0.5	1.03	0.92	0.83	0.53
**chemotherapy**	NO	317	0.93	0.67	1.39	0.1	1.1	0.68
	YES	178	0.7	0.099	0.93	0.74	0.66	0.087

Table S2. Subgroup analysis on the correlation of NOL7 expression and prognosis of lung cancer cases.

HR, hazard ratio; OS, overall survival; RFS,relapse free survival; AJCC, American Joint Committee on Cancer; FP, first progression; PPS, post progression survival; NA, not available data; *p* value less than 0.05 is shown in red.

Factor	Subgroup	Sample size	OS		PFS		PPS	
			HR	*p*	HR	*p*	HR	*p*
**Histology**	Endometrioid	62	5.77	0.076	5.77	0.00068	NA	NA
	Serous	1232	0.88	0.12	0.69	7.1e-07	0.77	0.0039
**Stage**	Stage 1	107	5.17	0.0018	4.49	0.012	NA	NA
	Stage 2	72	2.74	0.081	1.83	0.087	2.88	0.1
	Stage 3	1079	1.14	0.13	0.65	5.3e-08	0.79	0.012
	Stage 4	189	0.73	0.093	0.55	0.017	0.39	0.00017
**Grade**	Grade 1	56	1.59	0.32	2.79	0.055	NA	NA
	Grade 2	325	1.15	0.4	0.64	0.0078	0.72	0.085
	Grade 3	1024	0.88	0.14	0.73	0.00022	0.71	0.00074
	Grade 4	21	0.6	0.31	NA	NA	NA	NA
**TP53 mutation**	Mutated	516	0.68	0.0011	0.59	4.7e-06	0.61	0.00017
	Wild type	102	0.72	0.25	0.7	0.17	0.64	0.14
**Debulk**	optimal	802	1.55	2.4e-05	1.62	4.9e-06	0.78	0.0039
	suboptimal	536	0.84	0.096	1.3	0.021	0.66	0.002
**Chemotherapy**	Contains platin	1438	0.91	0.22	1.28	0.00062	0.75	0.0013
	Contains Taxol	821	1.14	0.22	0.72	0.00039	0.84	0.12
	Contains Taxol+platin	804	1.14	0.22	0.73	0.00057	0.83	0.11
	Contains Avastin	50	2.6	0.039	0.44	0.024	2.58	0.044
	Contains Docetaxel	108	0.55	0.08	0.78	0.41	1.19	0.56
	Contains Gemcitabine	135	1.36	0.17	0.61	0.021	0.81	0.34
	Contains Paclitaxel	248	0.71	0.13	0.81	0.22	0.66	0.15
	Contains Topotecan	119	1.57	0.034	0.78	0.29	1.39	0.11

Table S3. Subgroup analysis on the correlation of NOL7 expression and prognosis of ovarian cancer cases.

HR, hazard ratio; OS, overall survival; PFS, progress free survival; PPS, post progression survival; TP53, Tumor Protein P53; NA, not available data; *p* value less than 0.05 is shown in red.

Factor	Subgroup	Sample size	OS		FP		PPS	
			HR	*p*	HR	*p*	HR	*p*
**Gender**	Female	244	0.41	8.8e-0.5	0.51	0.0032	0.31	8.5e-07
	Male	566	0.48	2e-09	0.43	2.4e-07	0.36	8.2e-14
**Stage**	Stage 1	69	0	0.0035	0	0.0085	0.08	0.00022
	Stage 2	145	0.67	0.2	1.34	0.34	0.42	0.011
	Stage 3	319	0.49	6e-05	0.6	0.0082	0.32	1.1e-05
	Stage 4	152	0.56	0.013	0.51	0.0072	0.48	0.002
**Stage t**	t2	253	0.63	0.035	0.66	0.067	0.41	6.5e-05
	t3	208	0.68	0.034	0.75	0.12	0.44	0.00037
	t4	39	0.33	0.017	0.32	0.011	0.44	0.11
**Stage n**	n0	76	0	0.0015	0	0.0014	0.12	0.00022
	n1	232	0.41	0.00014	0.53	0.0017	0.27	4e-08
	n2	129	0.48	0.0041	0.65	0.05	0.38	2e-04
	n3	76	0.57	0.065	0.52	0.024	0.64	0.13
**Stage m**	m0	459	0.53	3.3e-05	0.53	4e-04	0.35	1.6e-11
	m1	58	0.39	0.0021	0.49	0.039	0.35	0.012
**HER2**	negative	641	0.48	1.3e-09	0.56	1.1e-05	0.33	1.1e-13
	positive	424	0.84	0.19	0.65	0.0082	0.43	8.7e-05
**Lauren classification**	Instestinal	336	0.45	7e-07	0.51	0.0012	0.31	3.3e-08
	Diffuse	248	0.61	0.0054	0.65	0.016	0.48	0.00041
	Mixed	33	0.34	0.052	0.31	0.038	NA	NA
**Differentiation**	Poorly	166	1.31	0.24	1.73	0.03	1.33	0.38
	Moderately	67	0.5	0.034	0.48	0.02	2.21	0.12
	Well	32	0.49	0.1	NA	NA	NA	NA
**Treatment**	Surgery alone	393	0.65	0.0078	0.73	0.08	0.4	8.2e-09
	5-Fu based adjuvant	157	1.72	0.0038	1.99	0.00019	1.28	0.19
**Perforation**	No	169	0.7	0.08	0.65	0.027	1.9	0.03

Table S4. Subgroup analysis on the correlation of NOL7 expression and prognosis of gastric cancer cases.

HR, hazard ratio; OS, overall survival; FP, first progression; PPS, post progression survival;

HER2, human epidermal growth factor receptor-2; NA, not available data; *p* value less than 0.05 is shown in red.

Factor	Subgroup	Sample size	OS		PFS		RFS		DSS	
			HR	*P*	HR	*P*	HR	*P*	HR	*P*
**Stage**	Stage 1	171	1.72	0.084	0.77	0.31	0.78	0.39	2.33	0.059
	Stage 2	86	0.62	0.24	0.69	0.29	1.19	0.6	2.01	0.24
	Stage 3	85	1.49	0.23	2.11	0.013	2.22	0.021	1.73	0.14
**Grade**	Grade 1	55	2.2	0.21	3.13	0.0029	3.3	0.012	0.55	0.34
	Grade 2	177	0.71	0.2	1.39	0.21	1.25	0.45	1.83	0.067
	Grade 3	122	1.79	0.065	0.6	0.06	0.54	0.037	2.04	0.14
**AJCC_T**	T1	181	1.83	0.043	0.85	0.51	1.25	0.41	2.49	0.026
	T2	94	0.56	0.12	0.78	0.39	1.2	0.56	0.62	0.39
	T3	80	1.73	0.1	1.83	0.054	1.94	0.065	1.78	0.15
**Gender**	Female	121	1.7	0.076	1.69	0.043	2.07	0.014	2.03	0.066
	Male	250	1.34	0.27	1.27	0.22	0.81	0.34	1.5	0.16
**Vascular invasion**	None	205	1.34	0.29	1.25	0.35	1.27	0.35	1.88	0.092
	micro	93	0.45	0.038	1.88	0.12	2.23	0.087	2.39	0.11
**Race**	White	184	1.92	0.027	1.88	0.0018	1.98	0.0035	1.95	0.026
	Asian	158	1.41	0.26	0.68	0.13	0.62	0.084	1.65	0.21
**Sorafenib treatment**	treated	30	3.22	0.036	2.18	0.05	1.74	0.24	3.22	0.036
**Alcohol consumption**	Yes	117	1.75	0.1	1.43	0.25	0.77	0.45	1.78	0.12
	none	205	1.39	0.18	1.49	0.87	1.6	0.066	1.87	0.044
**Hepatitis virus**	Yes	153	1.42	0.3	0.59	0.025	0.59	0.036	0.7	0.4
	none	169	1.57	0.051	1.92	0.0067	2.05	0.0048	2.41	0.0017

Table S5. Subgroup analysis on the correlation of NOL7 expression and prognosis of liver cancer cases.
HR, hazard ratio; AJCC，American Joint Committee on Cancer; OS, overall survival; PFS, progress free survival; RFS, relapse free survival; DSS, disease specific surviva; NA, not available data; *p* value less than 0.05 is shown in red.
